# Community Perception vs. Biochemical Confirmation: A Mixed-Methods Study on Water Quality From South India

**DOI:** 10.7759/cureus.19104

**Published:** 2021-10-28

**Authors:** Nagesh Ramya, Mahendra M Reddy, Prasanna B Kamath

**Affiliations:** 1 Community Medicine, Sri Devaraj Urs Medical College, Sri Devaraj Urs Academy of Higher Education and Research, Kolar, IND

**Keywords:** water analysis, water quality, sustainable developmental goal, sanitation, hygiene

## Abstract

Background

Community participation in water and sanitation is one of the prominent global indicators used to assess the achievement of water-related sustainable developmental goals. The participation by the community mostly depends on the way the community perceives their water source quality.

Objective

To measure the community perception regarding the quality of water concerning both drinking and domestic use and testing these perceptions with biochemical confirmation in a rural area of South India.

Methods

An exploratory sequential mixed-methods study design, comprising an initial cross-sectional quantitative study followed by qualitative field observations and in-depth interviews, was conducted to assess the community perceptions on the quality of water for drinking water and domestic use. Water samples were collected from 16 different sites and assessed for various biochemical parameters using standard guidelines. Quantitative data were reported using proportions and qualitative data was reported using categories and verbatim quotes.

Results

A total of 82 households were included in the survey. Among these households, 67% of the households used 'open dug well' as the source of their drinking water. None of the households was practising any purification method for drinking water. The community perceived the water quality to be good with no complaints but the perception of drinking water quality was based on sensorial factors like 'smell and colour' for drinking water and 'patches', and 'good lather and no stains' for domestic water use and not based on health or microbial quality of water*. *Biochemical analysis showed that biological oxygen demand and chemical oxygen demand were not within the prescribed standards in all the samples indicating considerable pollution. The deviation was more in 'stored samples' compared to 'source samples in all the water sources.

Conclusion

The study showed that community perception on water quality matched in a few aspects with biochemical confirmation but not all characteristics or beliefs were concurrent with biochemical analysis. There is a need to increase awareness regarding water, sanitation, and hygiene practices especially among women in the community, who are the primary stakeholders.

## Introduction

Ensuring access to water and sanitation for all is one of the goals (Goal 6) under the Sustainable Development Goals (SDGs) from the 2030 Agenda for Sustainable Development set up by the United Nations General Assembly. One of the eight targets set for this goal includes 'support and strengthen the participation of local communities in improving water and sanitation management' (Target 6B). The indicator used to measure the achievement of this target includes 'proportion of participation of local communities in water and sanitation management' [1,2].

The World Health Organization (WHO) emphasizes the role of community participation in the surveillance of water quality, as they are the primary stakeholders and also the ones to be affected first in case of decreased or bad water quality [3]. Perception of water quality plays an important role in the usage of water as that drives the usage of water in the community. The more important issue is the beliefs that lead to these perceptions by the community. As with any perceptions, these vary from region to region and could be based on the status of the community concerning literacy, socio-economic conditions, culture, and social factors.

The role of gaining the trust of those who use water was advocated by the International Water Association (IWA) in the year 2004 making this one of the primary goals for safe drinking water [4]. Most of the community perception as seen in studies from Western countries show that there was no scientific basis involved for drawing conclusions on water quality. The working group on 'preparing for the next generation of watershed management programmes and projects' in the year 2003 identified the 'role of scientific knowledge in public perceptions' as one of the innovative approaches to cope with emerging issues in water management [5].

Perceptions that truly reflect the actual quality of the water can help in the improvement of the overall quality of the water as well as its use by the community. Adversely, the wrong perceptions may interfere in the decision-making capacity of the community and lead to ineffective use of water. There is a paucity of literature that actually tests these perceptions with the actual quality of water especially in rural areas belonging to low and middle-income countries like India. Identifying the community perception of water quality is crucial not only for surveillance of water quality but also for the management of available water resources.

With this background, the current study was planned to measure the community perception regarding the quality of water with respect to both drinking and domestic use and in order to test these perceptions with biochemical confirmation.

## Materials and methods

Study design

We used an exploratory sequential mixed-methods study design with an initial cross-sectional quantitative study followed by qualitative field observations and in-depth interviews to know the community perceptions on the quality of water for drinking water and domestic use [6].

Quantitative component

Study Setting

A community-based cross-sectional study was conducted in a rural area of Kolar district (one village), Karnataka, South India. This is a village with naturally formed two clusters based on caste: Scheduled Caste (SC) / Scheduled Tribe (ST) and Other Backward Castes (OBCs).

Sample Size

The sample size was calculated keeping that at least 50% of the population will have access to good quality water; with absolute precision of 10%, the minimum required sample size was calculated to be 97 households (calculated using OpenEpi Version 3.01 [https://www.openepi.com]).

Study Procedure

A house-to-house survey was conducted from July to October 2018 to collect the socio-demographic details and details regarding domestic and drinking water use in the household. All the households in the village were included in the study. The details regarding the family socio-demographics and water-related characteristics were collected from an adult female of the household using a pre-tested semi-structured interview schedule after obtaining written informed consent. Households that were found locked were again attempted on the next two consecutive days and if still found locked on the third day the household was considered to be 'locked' and taken as non-response.

Biochemical Analysis

Water sample collection: The water samples were collected from 16 different sites from the rural community. A total of 10 samples were collected at the source - six samples were collected from each of the mini-water tanks placed in each street, one sample was collected from a dug well, and three samples were of water supplied through pipelines. A total of six stored water samples were collected - three samples of stored pipeline water, two samples of the stored mini water tank, and one sample of stored dug well water. All the water samples were collected in sterilized Borosil glass bottles. The type of sample collection done was 'grab sample'. The samples were transported to the laboratory within 90 minutes from the time of sample collection to ensure lesser contamination.

Water sample analysis: The following field observations were made - weather, temperature, colour, odour, and visible turbidity, visible effluent discharged and human activities around the water bodies were noted down at the time of sample collection. At the laboratory, the following parameters were estimated quantitatively using standard guidelines (see Supplement I) - Estimation of pH, temperature, dissolved oxygen (DO), biochemical oxygen demand (BOD), chemical oxygen demand (COD), total hardness of water, calcium (Ca2+), magnesium (Mg2+), total alkalinity of water, phosphate (PO4), sulphate (SO4), total dissolved solids (TDS), chlorine (Cl2) and fluorine (F). All results were interpreted with the WHO standards for drinking water [3,7]. All samples were checked for these parameters for three trials and the average of three readings was reported. A brief procedure is given separately in the table in the appendix for reference. 

Qualitative component

The observation was done regarding the water use and the surroundings around the water sources by the principal investigator and the findings were recorded in the field notes before conducting the in-depth interviews. The observation was made for a total of about four hours at different sources from 8 am to 12 pm for three days (alternate days) in a week.

An in-depth interview was done among women to assess their perception regarding the quality of water with respect to drinking water and domestic water use. The interview was conducted in their place of choice, and each interview lasted for about 15 to 20 minutes. A convenient sampling technique was used to obtain the sample and the interviews were carried out till data saturation was achieved. Interviews were also conducted among key informants - Accredited Social Health Activist (ASHA) and Auxiliary Nurse Midwife (ANM) - to know the practice and regularity of water analysis done in the village. The interview was conducted by the trained principal investigator, a 25-year-old woman. The co-investigator who was also trained in qualitative research acted as the note-taker. All interviews were conducted in the local language (Kannada) after obtaining written informed consent. The interviews were noted in the English language directly and were later transcribed into a Word document on the same day of the interview for analysis purposes. At the end of each interview, the investigator read out the notes and confirmed the validity of the same.

Ethics

The study protocol was approved by the Institutional Ethical Committee for Human studies of Sri Devaraj Urs Medical College, Kolar, Karnataka, India (SDUMC/KLR/IEC/20/2018-19). A written informed consent was obtained from all the study participants before the interview. Permission was also obtained from the village heads before the start of the study. This study was part of larger study which also assessed the water, sanitation, hygiene practice, and also the use of rain water harvesting in community [8,9]. 

Data entry and analysis

Quantitative

Data were single entered using Microsoft Excel and analyzed using SPSS Statistics, v. 20 (IBM Corp., Armonk, NY). Continuous variable like age was expressed using median and interquartile range (IQR). All biochemical parameters were expressed as mean with respective units. All categorical variables like gender, occupation, marital status, education, above/below poverty line status (APL / BPL), caste, and religion were expressed using frequency and proportions.

Qualitative

The field observations were written in field notes and results were expressed as 'statements'. Content analysis was done using an inductive process of grounded theory from the data collected. The results were analyzed under two broad domains or themes: (i) 'perception on quality of drinking water' and (ii) 'perception on quality of water used for domestic use'.

We used open coding wherein the data was divided into meaningful phrases. Categories were then identified to suit the central themes. All transcripts were entered in a Microsoft Word document. These transcripts were then manually coded into different categories by two different investigators. The results were reported using verbatim quotes under categories and verbatim quotes after reaching a consensus between investigators. The differences if any were cleared with the help of a third investigator. Investigator triangulation ensured the trustworthiness of qualitative analysis [10,11]. 

## Results

Quantitative

Out of the total 108 households enlisted a total of 82 households (76%) comprising 464 individuals were surveyed. The median (IQR) age was 27.5 (17.25 - 42.75) years. Out of the total 464 individuals surveyed, 238 (51.3%) were males, 262 (56.5%) were married, 187 (40.3%) had at least high school education, 251 (54.1%) were employed, 383 (82.5%) belonged to BPL, 350 (75.4%) belonged to SC/ST caste and all belonged to Hindu religion.

Out of the total 82 households surveyed, open dug wells were used as the source of drinking water in 55 (67.1%) households, and 45 (54.9%) households used steel vessels for storage of drinking water. None of the households were using long-handled ladles to draw water from the stored vessels nor practising any purification method for drinking water. With respect to domestic water use, 32 (39%) households used water from mini tanks for their domestic use. None of the households reported any contamination in water in the last year.

Qualitative

Field Observations

The water sources varied in both these communities. The SC/ST community had a dug well for their daily needs which had a proper concrete lining that prevented contamination with activities around the well. This well was situated at a distance of about half-to-one kilometre from the human dwellings and no other human activity around the well like bathing and washing clothes were seen. They also had access to piped water supply (municipal water) introduced about a year back with water supply for a particular duration of the day (one hour in the morning). These pipelines had no protection with higher chances of breakage and these lines were laid parallel and nearby the open drainage. The OBC community had a water supply from six mini tanks (each tank with two pipes) which were filled regularly with bore well water. Human activity was very rampant around these tanks with the washing of clothes. All these water tanks were situated at the side of the open drainage.

A total of eight in-depth interviews were conducted, three among the OBCs and five among the SC community. Details of the respondent characteristics are shown in Table 1.

**Table 1:** Demographic profile of respondents SC - Scheduled Caste, OBC - Other Backward Class, APL - Above Poverty Line, BPL - Below Poverty Line

Respondent	Age	Caste	Education	Occupation	Family size	Family type	Socio-economic status
1	35	SC	Illiterate	Unemployed	5	Nuclear	BPL
2	45	SC	Illiterate	Daily wage laborer	7	Three generation	BPL
3	30	SC	Primary school	Unemployed	2	Nuclear	BPL
4	28	SC	Secondary school	Daily wage laborer	4	Nuclear	BPL
5	25	SC	Secondary school	Daily wage laborer	6	Joint	BPL
6	45	OBC	Higher secondary school	Unemployed	8	Joint	APL
7	30	OBC	Secondary school	Unemployed	10	Three generation	APL
8	26	OBC	Higher secondary school	Unemployed	9	Three generation	BPL

The emphasis was to understand their perceptions on the quality of drinking water and the quality of water used for their domestic use. The two communities under study had different sources of water used for their daily living; while the OBC community depended on the piped water supply from mini water tanks (which had regular 24-hour supply) for all their daily needs, SC communities had access to both well water (single large hand-dug well with concrete safety wall) and piped water supply (irregular intermittent supply).

Perception on Quality of Drinking Water

When asked about the quality of drinking water, we found consistent answers in the two groups formed across caste. The results were categorized into four different categories 'regularity', 'smell and color', 'water source preference', and 'purification'. The verbatim quotes supporting these categories are given in Table 2.

**Table 2:** Categories and corresponding verbatim quotes denoting the major theme of 'perception on quality of drinking water'

Category	Verbatim quotes
I. Regularity	'Yes, the drinking water is fine and we get it regularly' (Respondent 6)
	'... I am happy with the quality of water and we don’t feel any necessity to store in large vessels…… the water is regularly available at a near distance' (smiles)( Respondent 7)
	'This tap water even if good it is not regular at all…. well water is available for us all the time and we can depend on it always…' (Respondent 5)
II. Smell and color	'Yes when they clean the tanks that time the water color seems muddy… so we catch and use it (i.e., water) for other purpose and after a time the water colour is normal…' (Respondent 8)
	'Oh yes yes!! When they clean tanks… the water smell mainly because they use bleaching powder to clean the tanks….' (Respondent 6)
	'We all drink well water, we like the taste and the water is so clear…' (Respondent 3)
III. Water source preference	'Yes well water is good for drinking….even though I go to work more of the times I prefer to fetch water from well than get it from nearby tap...' (Respondent 4)
	'We have been drinking the water from well from so long, my father, mother also were drinking from this well…. we feel it’s safe and….. see we are all so healthy' (smiles…) (Respondent 1)
IV. Purification	'Why should we boil water? It is very clean and free of any bad things…and the taste changes if we boil…' (Respondent 2)
	'…boiling makes the water tasteless…It would not be nice for drinking at all!!..' (Respondent 6)

Those who were OBC and thus getting water supplied through pipes, in general, were happy about the regularity and thus used to replace water frequently (most of them daily). The piped water supply in the SC community was irregular and not satisfactory.

When queried regarding the taste, colour, odour, and any visible particles in the drinking water (probes used: 'how is the colour of water', 'odour', 'taste' 'any visible particles'), we found there were few instances of change in colour of water and were attributed to the cleaning of tanks during that time.

All those belonging to the SC community used only their well water for drinking purposes. They had access to tap water but none of them were using it as a source of drinking water because of the belief that well water was more clean and superior in quality.

When probed regarding any methods adopted for purification and all of them responded that they did not feel the need for using any of the methods as they were happy with the quality of the water they drink and thus do not warrant any purification methods. When asked if they were any practice of purification for those who fall sick, some of them replied they would give lukewarm water. They would just heat water to make it hot enough to drink but not boil it as they felt it would alter the real taste of water. This thinking existed in both the castes irrespective of the source of water.

Perception on Quality of Water Used for Domestic Purposes

When asked about the water quality for domestic usage, both the communities believed that the quality of water they get is sufficiently good for washing clothes and utensils, bathing, and for their domestic purposes. The results were categorized into ‘accessibility’, ‘patches’, ‘preference’, and ‘good lather and no stains’ (see Table 3).

**Table 3:** Categories and corresponding verbatim quotes denoting the major theme of 'perception on quality of water used for domestic use'

Category	Verbatim quotes
	'We collect and store water in drums in the morning hours daily for washing and bathing… water is sufficient and regular' (Respondent 4)
I. Accessibility	'Water is good and we have access throughout the day so we just store in small vessels and refill as needed…..' (Respondent 7)
	'Both well water and pipe water is good for washing… for clothes I use pipe water as it is more accessible….' (Respondent 2)
II. Patches	'We see white patches in the vessels we use for boiling the water for bathing purposes…. I think it is common.. should we worry?' (Respondent 8)
III. Preference	'We are a family of two and we prefer well water for bathing…. I feel there is less hair loss this way…(chuckles)' (Respondent 7)
	'We have no issues with washing clothes….. lather is formed well and no other complaints….' (Respondent 6)
IV. Good lather and no stains	'We wash near the tank itself so we need not carry water to home… it gives good lather and no stains on clothes too…'
	'No stains are seen in clothes and the dirt also goes off easily…. no issue with water….' (Respondent 5)

The use of water for domestic purposes was decided based on accessibility as most of them felt there was no difference in the type of water. Few of the women responded as having a preference for well water for bathing purposes. Both types of water gave good lather and had no stains for washing clothes. The observation of washing clothes at the mini tanks was also confirmed in the interview. Most of them observed the formation of patches when water was boiled for bathing purposes but it did not cause any worry to them.

Findings From Key Informant Interviews

ANMs and ASHAs were interviewed to assess the practices adopted in the village with respect to water analysis. We found that the ASHA collected water from the source and also household for analysis during her monthly visits. The ASHA collected the water in a clean plastic bottle (these were bottles of used packaged drinking water). ASHA had no training in the collection of water samples but added that she had a briefing by the ANM regarding the water sample collection.

When enquired about the reports of analysis and dissemination of information to stakeholders it was observed that ASHA did not collect reports as it was not felt essential.

'If there was anything wrong (in the water analysis report) . . . they would have anyway informed me . . .'

Biochemical analysis

Field Level Analysis

All the water samples were found to be colourless, odourless, had no visible effluents or suspended particles, and were clear in appearance with no visible turbidity.

Laboratory Analysis

The mean values of the water samples segregated by source and storage are reported in table 4. Biological oxygen demand (BOD) and chemical oxygen demand (COD) were not within the prescribed WHO standards in all the samples indicating considerable pollution. The deviation was more in 'stored samples' compared to 'source samples' in all three water sources. With respect to source, the BOD and COD were comparatively better in the 'dug well sample' compared to the other two sources. The other biochemical parameters also showed the same result wherein the 'dug well sample' was comparatively better than the other two sources for both drinking and domestic use purposes (see Table 4).

**Table 4:** Report of biochemical analysis from water samples collected at source and stored samples

Biochemical Parameter studied (units)	Mini Water Tank		Dug Well		Pipeline	
Type of sample	At Source	Stored Sample	At Source	Stored Sample	At Source	Stored Sample
Number of samples	(6 samples)	(2 samples)	(1 sample)	(1 sample)	(3 samples)	(3 samples)
Dissolved oxygen (mg/dl)	5.6	5.6	6.5	6.1	6.1	5.6
Biological oxygen demand (BOD) (mg/dl)	7.7	8.2	7.3	8.4	8	8.4
Chemical oxygen demand (COD) (mg/dl)	6	6.1	5.3	6	5.6	5.7
Total Hardness (mg/dl)	156	156	131	138	138	149
Calcium (mg/dl)	85.6	86.3	76.7	85.7	78.6	78.1
Magnesium (mg/dl)	70.9	72	54.3	52	60	62.8
Total alkalinity (mg/dl)	195.6	200	133.3	146.7	113.3	117.8
Phosphate (mg/dl)	0.008	0.008	0.008	0.008	0.007	0.007
Sulphate (mg/dl)	0.03	0.04	0.03	0.04	0.03	0.03
Fluoride (mg/dl)	0.005	0.005	0.005	0.005	0.005	0.005
Chloride (mg/dl)	123.3	120	50	90	50	53.3
Total dissolved solids (mg/dl)	210.1	213.2	195.7	215	197.8	199.2
pH	7	7.2	7.2	6.5	7.1	7.2
Temperature (0C)	23.9	24.5	23	22.8	23.5	24

It was also noted that water from the mini-tank was 'hard' compared to 'moderately hard' in dug well and pipeline water according to WHO standards.

## Discussion

We conducted a mixed-methods study to explore the link between the perceptions on quality of water with actual biochemical parameters with respect to both drinking water and water for domestic use. The study area had an interesting feature of caste acting as a determinant for 'source of water'; the SC/ST community were using dug well water more frequently along with municipal piped water for their needs, whereas, the OBC community used bore well water available from mini tanks for their use.

Both the communities perceived that the water quality was good with respect to drinking and domestic use and had no major complaints regarding the quality. The quality perceived was mostly based on 'regularity', 'smell and colour' for drinking water and 'accessibility', 'patches', and 'good lather and no stains' for domestic water use. As the OBC had only one source of water there was no preference for the source; with respect to SC/ST community which had two sources of water, it was noted that all of them preferred 'dug well water' as the source of drinking water. The reasons quoted were mostly on the belief instilled by the elders that 'well water is good' and the use of the same well water by these households for generations. It was alarming to find that health and disease were not in the priority and it was also opinionated that all were healthy because they were drinking well water. This perception was also concurrent with the biochemical analysis which showed that well water had a better quality as per WHO standards for drinking water quality compared to the piped water supply. 

A study done in rural Maharashtra, India showed that water quality was perceived mostly based on smell, taste, and visible turbidity. Few also reported their experiences of ill health after consumption of water to decide on water quality. The study also reported that overall community piped water users' perceived water to be of better quality compared to those using bore well or dug well water [12]. Another study done across the banks of river Yamuna, stretching across India's capital city Delhi, showed that the majority of the responders rated the quality of water based on sensorial factors like smell, taste, and colour but not on health or microbial quality of water [13]. These study findings are concurrent to the findings of our study which deduced the perception was based on similar themes of sensorial factors than related to health factors.

Studies were done in other countries in university students to note the preference of bottled water use or other sources it was also noted that preference was based on accessibility, affordability, taste, colour, odour, turbidity and previous experience with water sources but also related to 'health reasons'. These 'health reasons' include 'ignorance' and 'mistrust' regarding the disinfection process, belief that 'tap water is only for irrigation, and thinking it will cause sickness' [14,15]. These studies were done on university students who are more educated ad cannot be compared to the general community awareness. The perceived quality must also be based on 'health reasons' and not only the sensorial factors of water as reported from studies in India.

The current study also highlighted an important aspect regarding the purification process of water after collection. It was felt by the entire community that there was no need for any purification as the water quality was good. This was more of the 'belief' that water was good as it was said by their elderly ('belief system' passed on by generations). The normally adopted household process like 'sieving using cloth' or 'boiling' seen in rural areas was also not in vogue in the study community. They opined that boiling reduces the 'taste' of water. None of the households used long-handled ladles to draw water from the stored vessels. These practices hampered the quality of water which was seen in the biochemical analysis which demonstrated that contamination was more in 'stored samples' compared to the 'source samples' (measured using BOD and COD). These findings call for the creation of health awareness regarding Water and Sanitation Hygiene (WASH) practices especially in rural areas using various approaches.

The key informant interviews from the current study showed that although regular water analysis was done there was no felt need by the health workers like ASHAs or ANMs to get access to the results and also the need for sharing the information with the community. A recent cluster-randomized trial in India showed that access to household water quality information actually led to safe WASH practices which included behaviour change in the purification of water [16]. Measurement at every household level and informing them of the results could not be a cost-effective strategy at the community level. The more feasible approach could be regular water analysis of the water at source and also the sample of household stored water with sharing of these results with the stakeholders like grass-root health care workers (ASHA, ANM, and Anganwadi workers) and also the community members. For this process to get into the system, there is a need to create awareness among these grass-root health care workers of the importance of gaining access and sharing the information with the community. The shared information on water quality could enhance or motivate people to adopt much safer WASH practices in the future.

We did not find any literature which studied the community perception regards to domestic water use. The current study showed that people perceive that domestic water is good or not based on the 'accessibility' to the water source, 'patches' formed on utensils when boiling water and also when washing utensils, and 'good lather and no stains' while washing clothes. These characteristics actually reflex the quality of water with respect to the hardness of the water. The current study showed that hardness was better for 'dug well' compared to 'mini tank' or 'pipeline water'. Similar to the drinking water, few women in the SC/ST community preferred well water to bathe compared to piped water due to the perception that it decreased hair loss. Although biochemical analysis showed dug well was better for domestic use people still used piped water supply mainly because of accessibility. As only women of the household get water from the source to household, and they are involved in domestic work they preferred the use of pipelined water which was more accessible. This study also vocals the concept of mainstreaming women in water management as envisaged by the United Nations Development Programme (UNDP) a decade ago [17].

The study has a few strengths. This is the first study from India which tried to link community perceptions with biochemical water analysis. We used a mixed-methods approach to explore the beliefs and perception of community rather than only questionnaire-based surveys which enabled us to identify factors that actually contributed to these perceptions. This study also gave insights into existing practices related to the use of water across different castes in an Indian village.

The study is not without limitations. Though this was the first study in the region to confirm the actual water quality and community perception, the analysis was restricted only to biochemical investigations and did not assess microbiological characteristics. The study findings' generalizability must be done with caution especially with regards to biochemical parameters, though the findings regarding how the community perceive water quality could be similar in other villages. 

## Conclusions

The study showed that community perception on water quality matched in few aspects with biochemical confirmation but not all characteristics or beliefs had concurrence with biochemical analysis. The community 'perceived' water quality is mostly based on sensorial factors like colour, odour, taste and also based on beliefs instilled by elders and not based on health concerns. These advocates for increased awareness regarding WASH practices and also the involvement of all stakeholders in improving water quality especially women in the community who are the primary stakeholders. Further research must focus on targeting interventions to improve the awareness of water, sanitation and hygiene among rural women in India.

## Appendices

The methods used to estimate the necessary biochemical parameters are detailed below (Table 5).

**Table 5:** Methods used in estimation of biochemical parameters mL – millilitre, N – normality, ^0^C - degree Celsius

Sl. No.	Estimated parameter	Brief procedure
1	pH	The pH of the water sample was checked using pHTestr10 (Thermo Fisher Scientific, Waltham, MA) in the laboratory.
2	Temperature	The temperature of each sample was recorded using a mercury thermometer (in Celsius) at the time of sample collection.
3	Dissolved oxygen	To 150 mL of the water sample, one mL each of manganese sulphate and alkaline iodide-azide solution was added. The contents were mixed well for 10 minutes and the brown precipitate was allowed to settle down for few minutes. Then to that one mL of concentrated sulphuric acid was added and mixed well. The treated sample was then titrated against sodium thiosulphate solution by adding four drops of starch indicator. The disappearance of the blue color was taken as the endpoint of the titration.
4	Biological oxygen demand (BOD)	150 mL of the water sample was taken in two reagent bottles each and was bubbled by blowing air into the sample. One mL each of phosphate buffer, magnesium sulphate, calcium chloride and ferric chloride was added and 0.5 mL of sodium hydroxide was added to get a pH of 7. One reagent bottle was kept for 5 days incubation at 200^0^C. The dissolved oxygen of one set was estimated immediately and dissolved oxygen of the another on day 5 of incubation.
5	Chemical oxygen demand (COD)	20 mL of the water sample was refluxed for two hours on water bath by adding a spatula full of mercury sulphate and 15 mL of 0.025N potassium dichromate solution, 35 mL of sulphuric acid silver nitrate reagent and glass beads. The contents were cooled after two hours of reflux and made up to 150 mL with distilled water. To this solution, three drops of ferrion indicator were added and titrated against ferrous ammonium sulphate. The colour change from blue-green to wine-red was taken as the endpoint of the titration.
6	Total hardness of water	To 25 mL of the water sample 0.5 mL each of buffer solution and sodium sulphate were added. A pinch of ferrichrome black T indicator was added and the contents were titrated against EDTA solution. The colour change from wine red to blue colour was taken as the endpoint of the titration.
7	Calcium	To 25 mL of the water sample one mL of sodium hydroxide solution was added and titrated against EDTA solution by adding a pinch of calcon indicator. The colour change from pink to blue was taken as the endpoint of the titration.
8	Magnesium	The value of magnesium was determined by subtracting the value of calcium from the total hardness of water.
9	Total alkalinity of water	Phenol alkalinity: To 25 mL of water sample, four drops of phenolphthalein indicator was added and titrated against hydrochloric acid. The disappearance of pink colour was taken as the endpoint of the titration. Methyl alkalinity: To 25 mL of the water sample three drops of methyl orange indicator were added and contents were titrated against hydrochloric acid. The colour change from orange to pink colour was taken as the endpoint of the titration. The total alkalinity of water was determined by adding the values of phenol alkalinity and methyl alkalinity.
10	Phosphate	10 mL of the water sample, blank and standard were taken in three different test tubes. To this 0.5mL of ammonium molybdate solution was added followed by five drops of stannous chloride solution. The optical density of the blue colour solution was taken at 690 nm after 10 minutes.
11	Sulphate	10 mL of sample and 10 mL of the standard sulphuric acid solution (0.02 N) were taken in separate test tubes. To this 0.5 mL conditioning reagent was added and mixed well. Later a pinch of barium chloride was then added and optical density was measured at 420 nm after four minutes.
12	Total dissolved solids	A measured volume of filtered water sample was taken in a pre-weighted beaker. The water was allowed to evaporate at 98^o^C on a hot plate. After the complete evaporation of water, the beaker was allowed to cool to room temperature and the weight of the beaker was measured. The total dissolved solids were calculated by subtracting the weight of the post and pre-weighted beaker values.
13	Chloride	10 mL of water sample was taken in a test tube and three drops of chromate solution was added and mixed thoroughly. To the obtained yellow-coloured solution, silver nitrate solution was added drop-wise with continuous mixing till the solution turns brick red. The number of drops of silver nitrate solution added was multiplied by 10 to get the amount of chloride in the water sample. The quantity of chloride was measured using Kit (supplied by ESSAR Laboratories and Research Centre) to analyze water in the fields.
14	Fluoride	To the five mL of water sample taken in the test tube, 10 drops of fluoride reagent was added and incubated for five minutes. The color obtained was compared with the standard color chart for the amount of fluoride present in the water sample. The quantity of fluoride was measured using kit (supplied by ESSAR Laboratories and Research Centre) to analyze water in the fields.
